# Time of Day and Training Status Both Impact the Efficacy of Caffeine for Short Duration Cycling Performance

**DOI:** 10.3390/nu8100639

**Published:** 2016-10-14

**Authors:** James C. Boyett, Gabrielle E. W. Giersch, Christopher J. Womack, Michael J. Saunders, Christine A. Hughey, Hannah M. Daley, Nicholas D. Luden

**Affiliations:** 1 Human Performance Lab, Department of Kinesiology, James Madison University, Harrisonburg, VA 22807, USA; boyettjc@dukes.jmu.edu (J.C.B.); gierscge@dukes.jmu.edu (G.E.W.G.); womackcx@jmu.edu (C.J.W.); saundemj@jmu.edu (M.J.S.); 2 Department of Chemistry, James Madison University, Harrisonburg, VA 22807, USA; hugheyca@jmu.edu (C.A.H.); daleyhm@dukes.jmu.edu (H.M.D.)

**Keywords:** exercise time of day, caffeine supplementation, training history, diurnal, training status

## Abstract

This project was designed to assess the effects of time of day and training status on the benefits of caffeine supplementation for cycling performance. Twenty male subjects (Age, 25 years; Peak oxygen consumption, 57 mL·kg^−1^·min^−1^) were divided into tertiles based on training levels, with top and bottom tertiles designated as ‘trained’ (*n* = 7) and ‘untrained’ (*n* = 7). Subjects completed two familiarization trials and four experimental trials consisting of a computer-simulated 3-km cycling time trial (TT). The trials were performed in randomized order for each combination of time of day (morning and evening) and treatment (6mg/kg of caffeine or placebo). Magnitude-based inferences were used to evaluate all treatment effects. For all subjects, caffeine enhanced TT performance in the morning (2.3% ± 1.7%, ‘very likely’) and evening (1.4% ± 1.1%, ‘likely’). Both untrained and trained subjects improved performance with caffeine supplementation in the morning (5.5% ± 4.3%, ‘likely’; 1.0% ± 1.7%, ‘likely’, respectively), but only untrained subjects rode faster in the evening (2.9% ± 2.6%, ‘likely’). Altogether, our observations indicate that trained athletes are more likely to derive ergogenic effects from caffeine in the morning than the evening. Further, untrained individuals appear to receive larger gains from caffeine in the evening than their trained counterparts.

## 1. Introduction

Caffeine use in sport is widespread due to its reputed performance benefits. There is consistent evidence that caffeine enhances cycling performance in events lasting longer than a few minutes [1,2,3,4,5]. While not unanimous [6,7,8], caffeine intake can also improve peak anaerobic power and speed [9,10,11] as well as peak muscle function (strength, power, and endurance) under certain conditions [6,7,12,13]. Although caffeine has the capacity to improve physical performance, there are a number of unresolved factors that may impact the magnitude of the effect of caffeine, such as time of day and training status.

Only two studies have investigated the potential interaction between time of day and caffeine on performance outcomes, and both suggest that the value of caffeine is heightened in the morning. In the first study, caffeine increased peak squat power in the morning but not in the evening [13]. Caffeine appeared to compensate for underperformance in the morning placebo trial such that squat power was elevated to levels observed in both evening trials (caffeine and placebo). We recently investigated whether time of day influenced the effects of caffeine on cycling performance, using a post-hoc analysis in which cyclists who completed trials early in the day (prior to 10 a.m.) were compared to those who performed later in the day (after 10 a.m.) [5]. In line with Mora-Rodríguez et al., caffeine ingestion improved performance among subjects that completed their trials early in the day but had an unclear effect on performance in those who performed later trials. Based on these preliminary results, the primary purpose of the present study was to use a crossover design to test the hypothesis that caffeine would elicit larger improvements in 3-km time trial (TT) performance in the morning compared to the evening.

Like time of day, training status may also mediate the magnitude of caffeine’s ergogenic effect. A 2010 meta-analysis indicated that caffeine tended (*p* = 0.08) to enhance muscle endurance in untrained more so than trained subjects [14]. However, this conclusion was largely reached by comparing effect sizes derived from studies with trained subjects to other studies with untrained individuals. Regardless of the performance measure, we are aware of only four investigations that included both trained and untrained subjects in the same experimental design, the first of which reported that caffeine improved 100 m swim performance more so in trained than untrained swimmers [10]. Though this is in contrast to the meta-analysis, it may not be fair to use swimming as a model to determine the effects of training status, as the technical nature of swimming mechanics likely made it difficult for the untrained swimmers to take full advantage of potential improvements in whole muscle function. The only other study to compare trained and untrained subjects in the same design, that also observed caffeine-induced improvements in performance, reported that untrained and trained subjects experienced similar improvements in 10-km cycling performance [15], which again is in contrast to the prior mentioned meta analysis. The other two studies concluded that training status had no effect on time-to-fatigue [16] or peak strength [17], although there was no main effect of caffeine in either study. The lack of a significant ergogenic effect of caffeine in these studies (i.e., experimental models that did not detect a beneficial effect of caffeine) makes it impossible to tease out the impact of training levels. We recruited participants that were accustomed to cycling exercise and ultimately enrolled subjects that had a wide range of cycling experience and fitness levels. This allowed us to examine a separate factor (other than time of day) that may alter the magnitude of benefit conferred by caffeine ingestion. Specifically, in addition to time of day, we tested the hypothesis that untrained cyclists would receive more of a performance benefit from caffeine compared to their trained counterparts.

The outcomes of this investigation have marked practical relevance. Athletes and coaches make training/competition decisions based on risk and reward. It is therefore worthwhile to establish whether or not time trial performance is differentially impacted by time of day and/or training status, as this will instruct best practices for caffeine use as an ergogenic aid. There can be downsides to caffeine consumption, particularly in the evening. For instance, caffeine intake later in the day can interfere with quantity and quality of sleep [18], thereby possibly impairing recovery from heavy exercise [19] and subsequent performance [20]. Our collective hypothesis was that trained subjects supplementing with caffeine in the evening will experience the least improvement in performance and therefore should reconsider caffeine as an ergogenic aid late in the day.

## 2. Materials and Methods

### 2.1. Subjects

Twenty-two healthy male subjects from James Madison University and the surrounding area volunteered for the study. Two subjects withdrew for reasons unrelated to the study, resulting in complete data from eleven trained and nine untrained cyclists. Descriptive data are shown in Table 1. Subjects were required to have performed, at minimum, either “occasional” cycling (one day/month) for the untrained cyclists or “consistent” cycling (four days/week) in their weekly exercise routine over the past three months for trained cyclists. Cycling frequency and duration were self-reported. Trained and untrained cyclists were determined by the number of hours cycling per week, with comparison based on the top (trained) vs. bottom (untrained) tertiles. The categorization of untrained and trained subjects is generally supported by individual peak oxygen consumption (VO_2peak_) values (Table 1). The notable exception is that one ‘untrained’ subject possessed a VO_2peak_ of 61.3 mL·kg^−1^·min^−1^. However, this subject was only performing 1.5 h of weekly cycling. Subjects provided information about their resistance training routines and this information was used as a covariate for all analyses (data reported in Table 1). Subjects were informed of the experimental procedures and risks prior to giving written consent. The study was approved by the James Madison University Institutional Review Board (IRB #15-0559). We also implemented a questionnaire asking about caffeine habits (coffee, tea, soda, chocolate, etc.); daily caffeine intake was calculated by assigning typical caffeine values to each respective item. Caffeine levels are reported in Table 1. Only one subject regularly consumed >300 mg/day, the previously established benchmark for ‘high’ caffeine intake (400 mg/day). Therefore, any differences in caffeine intake between subjects likely had a negligible impact on our performance outcomes.

### 2.2. Cardiovascular Fitness Testing

Following height and body weight measurements, subjects performed an incremental exercise test to exhaustion on a bicycle ergometer (Velotron, Racermate, Inc., Seattle, WA, USA) to determine peak oxygen consumption (VO_2peak_). The test began at a workload of 100 W (untrained) or 150 W (trained), and was increased by 25 W every minute until volitional fatigue. Metabolic measurements were assessed using a Moxus Modular Metabolic System (AEI Technologies, Pittsburgh, PA, USA) throughout the test and VO_2peak_ was determined by the highest 30-s mean oxygen uptake.

### 2.3. Experimental Design

A randomly counterbalanced, double blind, placebo controlled design was implemented to compare the effects of the four different treatment conditions. Subjects performed four trials: two morning trials starting between 6:00 a.m. and 10:00 a.m. (but with consistent starting times within each subject), and two evening trials starting between 4:00 p.m. and 8:00 p.m., with an eight-hour minimum separation between morning and evening start times for each subject. During the experimental trials, subjects ingested a capsule one hour prior to exercise containing either 6 mg/kg body weight anhydrous caffeine or all-purpose flour (placebo). Only ad libitum water consumption was permitted following capsule consumption. The four treatment conditions were designated as: 1 Morning placebo (AM_PLA_); 2 Morning caffeine (AM_CAF_); 3 Evening placebo (PM_PLA_); and 4 Evening caffeine (PM_CAF_).

### 2.4. Performance Trials

Each subject performed six exercise trials (two familiarization trials followed by four experimental trials) on both an isokinetic dynamometer (Biodex Multi-Joint System—PRO, Biodex Medical Systems, Inc., Shirley, NY, USA), and cycle ergometer, with 6 (2.5–17) days between each experimental trial. Venous blood samples were obtained immediately upon arrival to the laboratory and again prior to exercise (one-hour following capsule consumption). Subjects then began each trial with a 5-min treadmill warm-up at 3.5 mph. Following the warm-up, subjects completed two sets of four leg extension repetitions on an isokinetic dynamometer (two warm up repetitions followed by two peak torque measurements) at 30 degrees/s with the right leg. Each set was separated by 60 s. This protocol was repeated at 120 degrees/s and 240 degrees/s, respectively (grand total of 24 repetitions; 12 total warm-up repetitions (4 at each speed) and 12 total maximum repetitions (4 at each speed)). After a ~3 min transition, subjects performed a flat 3-km time trial on the cycle ergometer. The familiarization trials were identical to the experimental trials, with the exception of the supplementation protocol. Cycling power output (and consequently cycling velocity) was self-controlled by adjusting both resistance on the flywheel using a simulated gear shifter and pedaling cadence. Subjects were instructed to treat each trial as a competition prior to the beginning of each trial, but subjects did not receive verbal feedback or encouragement from the investigators during testing. Further, no visual feedback from the time trial was provided, with the exception of elapsed distance. 3-km time trial time was used as the performance measure.

### 2.5. Serum Caffeine Levels

Blood samples were obtained from the antecubital vein. After 30 min of coagulations, samples were centrifuged at 2500 rpm for 15 min. Serum was stored at −80 °C until analysis. Serum caffeine levels were subsequently determined via mass spectrometry.

#### 2.5.1. Sample Preparation for Liquid Chromatography/Mass Spectrometry Analysis

Serum samples were stored at −80 °C prior to extraction. 200 μL of serum was extracted by vortexing with 5 mL of ethyl acetate for 5 min. The extract was then centrifuged for 10 min at 4000× *g* to separate the organic and aqueous layers. The top ethyl acetate layer was transferred to a tube, the extraction repeated and the organic fractions combined. The extract was then lyophilized in a CentriVap (Labconco, Kansas City, MO, USA) and reconstituted in 200 μL of 96:4 water:methanol for quantitation by LC/MS.

#### 2.5.2. LC/MS Analysis

An Agilent 1290 ultra-high performance liquid chromatograph (UHPLC) coupled to a 6224 time of flight mass spectrometer (TOF MS) (Agilent Technologies, Santa Clara, CA, USA) was used to separate caffeine from other metabolites and measure its concentration in the serum extracts. Gradient elution with an Agilent Zorbax Eclipse Plus C18 column (2.1 mm × 150 mm, 1.8 μm particles) held at 35 °C was performed with mobile phase A (water, 0.1% v/v formic acid) and B (acetonitrile, 0.1% v/v) at 0.45 mL/min. as follows: B was held at 4% for 7 min and increased to 70% by 12 min. At 14.5 min the gradient was returned to the initial conditions. Five microliters of serum extract were injected in duplicate. Caffeine was ionized by positive ion electrospray (ESI) as follows: capillary, +3500 V; drying gas, 350 °C and 10 L/min; nebulizer 30 psig. Mass spectral data was acquired in profile and centroid mode at 3 specta/s over 100–1700 m/z. TOF ion optics were: fragmentor, 115 V; skimmer, 65 V and octopole retardation factor V_p-p_, 750 V. An internal reference mass (IRM) solution (purine and HP-921, Agilent Technologies, Santa Clara, CA, USA) was delivered to the ESI source to ensure high mass accuracy (<15 ppm).

A caffeine stock solution (1000 ppm, water) was serially diluted to yield a minimum of seven calibration levels that ranged from 0.01 to 20 ppm. Agilent’s Mass Hunter Quantitative Analysis software (B.06) (Agilent, Santa Clara, CA, USA) was used to generate external calibration curves and calculate the concentrations of caffeine in ppm.

### 2.6. Dietary and Exercise Control

Subjects were provided with instructions for recording food intake so dietary intake could be replicated across trials. All subjects recorded food intake for 24 h prior to all experimental trials. Subjects were provided with a copy of food records from the 24 h preceding the initial experimental trials to be used to facilitate dietary replication for the 24-h time period preceding subsequent trials. Subjects were also instructed to abstain from any alcohol (24 h), caffeine (12 h), and food intake (4 h; post-absorptive state) prior to each experimental trial. Our intent was to collect performance data in the morning and evening under similar feeding conditions. The most feasible way to accomplish this was to study subjects in a post-absorptive state, so as to avoid early waking and feeding prior to the morning trial. However, this leads to discrepancies in fasting duration prior to the morning and evening trials; the morning trials were conducted after an overnight fast (~7–10 h of fasting) whereas the evening trials were performed after a 4-h fast. While it is conceivable that this variance could impact performance, performance (both strength and 3-km TT) was virtually identical between the morning and evening under placebo conditions, suggesting that any error variance due to different fasting durations was likely negligible. Subjects were instructed to maintain consistent exercise habits between trials and to abstain from any heavy and/or unaccustomed exercise 48 h prior to each experimental trial. Subjects submitted physical activity logs for verification.

### 2.7. Statistical Analysis

All data were log transformed to diminish the effects of nonuniformity. Magnitude-based inferences about the data were derived using methods described by Hopkins and colleagues [21]. A previously established ‘smallest worthwhile change’ in performance was used as the threshold value for a substantial treatment effect (separate treatment conditions vs. placebo) [22]. The smallest worthwhile change in performance was defined as 0.3 × the within-subject variability of a similar group of cyclists previously studied in our laboratory [5] (Coefficient of Variation = 2.7% for time) which translates to a difference of 0.8% or 2.4 s in the current project [23]. As recommended by Hopkins, for the isokinetic data, 0.2 × SD of the AM_PLA_ trial was used to determine smallest worthwhile change [22]. The coefficient of variation for peak strength measurements (derived from placebo conditions) was: 3.9% at 30 degrees/s, 3.2% at 120 degrees/s, and 4.6% at 240 degrees/s. The coefficient of variation for 3-km TT performance was: 1.1% for all subjects, 1.1% for trained, and 0.8% for untrained.

A published spreadsheet [24] was then used to determine the likelihood of the true treatment effect (of the population) reaching the substantial change threshold (0.3 × CV); these were classified as <1% almost certainly no chance, 1%–5% = very unlikely, 5%–25% = unlikely, 25%–75% = possible, 75%–95% = likely, 95%–99% = very likely, and >99% = almost certain. If the percent chance of the effect reaching the substantial change threshold was <25% and the effect was clear, it was classified as a ‘trivial’ effect. If 90% confidence intervals included values exceeding the substantial change threshold for both a positive and negative effect, effects were classified as unclear (>5% chance of reaching the substantial threshold for both a positive and negative effect). To test the effects of time of day, the outcomes derived for each group using the spreadsheet mentioned above [24] were compared using a second spreadsheet [25]. Likewise, the effects of training status were compared using this same method. All data reported as mean ± 90% Confidence Interval unless noted otherwise.

We estimated the statistical power of our experimental design using a publicly available spreadsheet created for magnitude-based inferences [26]. Data derived from a subset of male subjects (*n* = 24) using a similar measurement protocol in our laboratory was used to estimate within-subject variability [5]. With a sample size of 20, the current design and statistical methods had the statistical power of 0.99 to detect changes in time trial performance of 1.5% and 0.7 to detect a performance change of 0.8%. For leg extension an effect of 4.05% (smallest meaningful effect derived from 0.2 × within subject standard deviation under placebo conditions) could be detected with a power of 0.96. The between subject comparisons (trained vs. untrained) were associated with low power thereby increasing the likelihood of making a type II error. However, we detected magnitude-based differences in 3-km TT performance (caffeine vs. placebo) between trained and untrained subjects and these data are reported; peak strength data specific to each training group are omitted because of the lack of power and lack of clear statistical outcomes.

## 3. Results

### 3.1. Serum Caffeine Levels

Serum caffeine levels in AM were: All Subjects—Pre 0.7 ± 1.3 ppm, Post 13.8 ± 2.4 ppm; Trained—Pre 0.6 ± 0.9 ppm, Post 13.1 ± 2.0; Untrained—Pre 0.2 ± 0.3 ppm, Post 13.6 ± 2.3 ppm. Caffeine levels in PM were: All Subjects—Pre 0.7 ± 0.8, Post 14.7 ± 3.1 PPM; Trained—Pre 0.6 ± 0.7 ppm, Post 13.1 ± 3.9 ppm; Untrained—Pre 0.6 ± 0.5 ppm, Post 15.0 ± 2.8 ppm. There were no differences between trained and untrained subjects, nor were there any differences between AM and PM caffeine levels following caffeine ingestion.

### 3.2. The 3-km Time Trial Performance

#### 3.2.1. All Subjects

**Figure 1:**
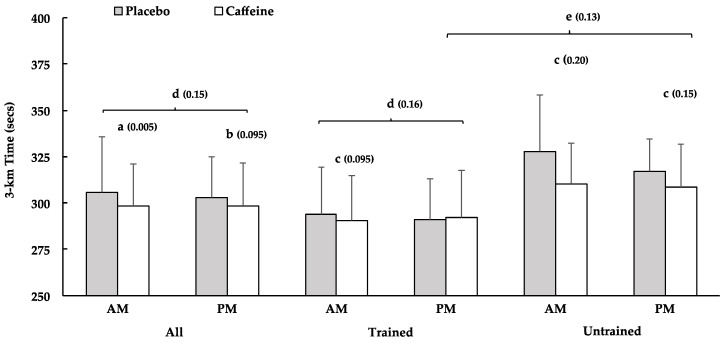
The 3-km Time Trial Performance. Bars depict mean finishing time in seconds (±SD). AM, morning; PM, afternoon; (**a**) ‘very likely’ faster than PLA; (**b**) ‘possibly’ faster than PLA; (**c**) ‘likely’ faster than PLA; (**d**) ‘likely’ different response to caffeine between AM and PM; (**e**) ‘likely’ different response to caffeine between Trained and Untrained in PM. *p*-values derived from pairwise comparisons are displayed in parentheses.

**Figure 2:**
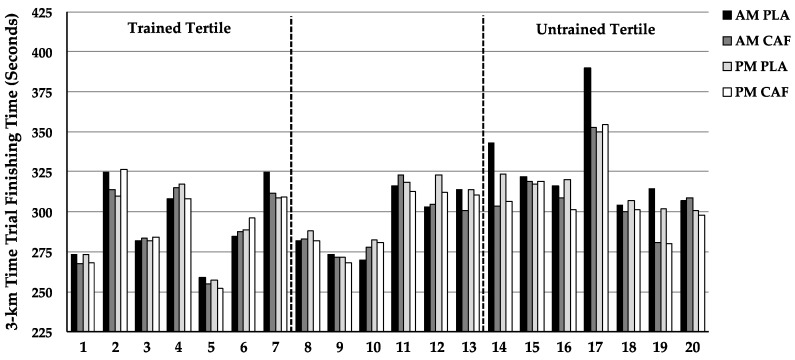
Individual 3-km Time Trial Performances. AM, morning; PM, afternoon; PLA, placebo; CAF, caffeine.

All 3-km performance data are displayed in Figure 1. Individual performance data are displayed in Figure 2. In all subjects, AM_CAF_ 3-km time trial performance (3-km TT) was ‘very likely’ better than AM_PLA_ (2.9% ± 1.7%), while PM_CAF_ ‘possibly’ improved performance vs. PM_PLA_ (1.1% ± 1.1%)*.* AM_CAF_ ‘likely’ improved 3-km TT performance to a greater extent than PM_CAF_ (1.7% ± 2.0%) when compared to the respective placebo condition (PLA).

#### 3.2.2. Trained Subjects

AM_CAF_ performance was ‘likely’ faster than AM_PLA_ (1.8% ± 1.9%), whereas caffeine’s effect was ‘unclear’ in the evening (PM_CAF_ vs. PM_PLA_: −1.0% ± 3.1%). Additionally, AM_CAF_ ‘likely’ improved performance more than PM_CAF_ (AM_CAF_ vs. PM_CAF_: 2.8% ± 3.4%), when compared to PLA.

#### 3.2.3. Untrained Subjects

AM_CAF_ and PM_CAF_ ‘likely’ improved time trial performance vs. AM_PLA_ (5.5% ± 8.0%) and PM_PLA_ (3.2% ± 3.8%), respectively. The time of day (AM vs. PM) comparison was ‘unclear’.

#### 3.2.4. Training Status

It was ‘unclear’ whether trained or untrained benefited more from caffeine in the AM condition, but untrained subjects ‘likely’ benefited more from caffeine supplementation than trained in the PM condition (trained: −1.0% ± 3.2%, untrained: 3.2% ± 3.8%, AM_CAF_ vs. PM_CAF_: 4.2% ± 4.5%).

### 3.3. Peak Muscle Torque

All peak skeletal muscle torque data are presented in Table 2. Knee extension torque at 30 degrees/s (30EXT) was ‘possibly’ improved by caffeine in PM when compared to PM_PLA_, but all other PM measures were ‘likely’ trivial. PM Caffeine ‘possibly’ increased PM_CAF_ torque more than AM_CAF_ torque in the 30EXT condition when compared to PLA. All other time of day comparisons were ‘trivial’ or ‘unclear’.

Bars depict mean finishing time in seconds (±SD). (a) ‘Very likely’ faster than PLA; (b) ‘possibly’ faster than PLA; (c) ‘likely’ faster than PLA; (d) ‘Likely’ different response to caffeine between AM and PM; (e) ‘Likely’ different response to caffeine between Trained and Untrained in PM. *p*-Values derived from pairwise comparisons are displayed in parentheses.

Data are reported as individual 3-km finishing times under all four experimental conditions, grouped by training tertiles. Numbers below the horizontal axis (x-axis) represent each individual subject.

## 4. Discussion

The purpose of the current study was to investigate how the benefit of caffeine for 3-km cycling TT performance was influenced by time of day and training status. Caffeine enhanced 3-km TT performance more in the morning than in the evening (all subjects and trained subjects). Caffeine also improved cycling performance among untrained subjects in the morning and evening, whereas the benefit for trained subjects was ‘likely’ in the morning and ‘unclear’ in the evening. Further, caffeine intake enhanced 3-km performance more among untrained- than trained subjects, in the evening. Secondarily, we assessed peak muscle strength at three separate angular velocities prior to the time trials. Caffeine has been shown to increase peak strength [6,7,12,13,27] and there is some evidence that strength may contribute to the ergogenic properties of caffeine for cycling performance [28]. Therefore, we measured peak strength in an attempt to provide some physiological insight into the time trial outcomes. However, caffeine only increased strength at the slowest velocity (30 degrees/s) in the evening, which does not align with the TT performance results. This suggests that the gains in time trial performance were not mediated by improvements in strength.

Consistent with our general hypothesis, caffeine enhanced 3-km TT performance among trained subjects in the morning but not the evening. This supports results from a recent study, in which we reported that caffeine supplementation elicited the largest improvements in 3-km cycling TT performance among subjects that completed trials prior to 10:00 a.m. [5]. Importantly, prior observations made in strength-trained participants that caffeine elevates performance in the morning but not the evening [13] can now be extended to include longer sustained efforts. To our knowledge there are no other data from which to directly compare our findings.

The scant information on this topic also makes it difficult to provide a well-founded explanation for why caffeine appears to deliver a more pronounced benefit in the morning. We suspected that the time of day differences in performance could be related to varying rates of caffeine metabolism throughout the day. Cytochrome P450 1A2, the enzyme responsible for caffeine metabolism, has been shown to have higher activity levels during sleeping hours and directly after waking, when compared to the rest of day [29]. Considering that caffeine metabolites appear to be more potent than caffeine itself, faster caffeine metabolism could lead to a higher concentration of metabolites in the morning thereby delivering a stronger effect [30]. However, this was not the case in the current study. Caffeine levels were virtually identical between AM and PM trials (reported in Section 3.1). An alternative hypothesis is that the greater gains with caffeine in the morning are related to slower time trial performances in the morning compared to the evening, in the absence of caffeine. Though the physiology is largely unknown, there is good evidence that somatic control and physical performance (peak muscle strength, power, and swimming) can be impaired in the morning compared to the evening [20,31,32,33], perhaps providing an opportune time to utilize performance enhancing agents. This idea is supported by Mora-Rodriguez et al. where physical performance was worse in the morning compared to the evening, and caffeine raised morning performance to the levels achieved in the afternoon trials. The current data does not seem to support systematic somatic deficits in the morning, as only 9 of 20 subjects (2 of 7 trained tertile and 5 of 7 untrained tertile) performed slower in the AM_PLA_ than the PM_PLA_. However, 5 of these 9 subjects (1 trained; 4 untrained) had much slower times under AM_PLA_ conditions, which had a large effect on the overall outcomes (i.e., larger gains in AM vs. PM). These slower times may represent a true time of day effect or may reflect individual circadian rhythms. Unfortunately, we do not have chronotype data from which to test this possibility.

While training status did not affect the response to caffeine in the morning, the untrained tertile did experience a more favorable response to caffeine than trained subjects in the evening. This aligns with a recent meta-analysis on this topic that concluded that caffeine tended to improve muscle endurance more in untrained than in trained subjects [14]. The current data are an important addition to our understanding since, as highlighted in the introduction, this conclusion was largely deduced by comparing effect sizes derived from separate studies conducted on trained vs. untrained cohorts. The differential impact that training status had on the caffeine benefits in the evening is a function of both the lack of improvement among the trained subjects and a ‘likely’ beneficial effect among untrained subjects. The physiological mechanisms responsible for this result are unknown and beyond the scope of this investigation. However, the concentration of adenosine receptors (the presumed primary target of caffeine) do appear to be higher in trained compared to untrained individuals [34]. And though highly speculative, the higher concentration of adenosine receptors may increase tissue sensitivity to any given concentration of adenosine, thereby requiring larger doses of caffeine to elicit a desirable effect. This may especially be an issue when the effects of caffeine are expected to be relatively small (i.e., the evening).

The current project revealed that caffeine’s effect on 3-km TT performance was partially mediated by time of day and training status. However, peak muscle torque was largely unaffected by caffeine except ‘possibly’ at the slowest speed of contraction (30 degrees/s). There is some precedent for null strength findings [35,36,37], but most of the literature suggests that peak muscle function is heightened with caffeine [6,7,12,13,27]. Interestingly, as angular velocity increases, so do the number of trivial outcomes, indicating that movement velocity may impact the effects of caffeine. This could possibly be related to caffeine’s role as an adenosine antagonist, a mechanism responsible for its ergogenic effects [38]. Adenosine receptor density has been shown to be greater in slow-twitch muscle fibers [39]. However, higher movement velocities require a greater reliance on force output (and power) from fast twitch fibers due to reductions in slow twitch fiber power production secondary to shifting the velocity × power curve to the right [40]. Therefore, at the higher movement velocities, it is possible that the fiber type most responsive to caffeine supplementation (slow twitch fibers) would contribute a smaller proportion to whole muscle power output, resulting in a smaller measurable effect of caffeine. This would explain why no ergogenic effects of caffeine were observed for peak strength at speeds greater than 30 degrees/s. In support of this idea, Jacobson et al. [41] reported improvements in isokinetic knee extension strength with caffeine consumption which were greater at slower movement speeds.

## 5. Conclusions

The primary weaknesses of the current study include the relatively small sample size, the lack of mechanistic insight (RPE, muscle pain, etc.), and as discussed in Section 2.6, the markedly different fasting durations preceding the morning and evening trials. Specific to the latter, it seems possible that the different fasting durations preceding the morning and evening trials could have influenced performance in both placebo and caffeine conditions. However, performance was virtually identical across placebo trials (morning vs. evening). Further, despite evidence that feeding status can influence the pharmacokinetics of caffeine ingestion [42], caffeine levels were similar in both caffeine conditions, suggesting that the 4 h of fasting, regardless of duration, likely leads to similar rates of caffeine absorption/metabolism. Notwithstanding these potential issues, the findings of this study support the idea that time of day and training status influence caffeine ergogenics and that these are probably not mediated by peak strength. This suggests that caffeine may be a suitable supplement for use during morning competition, but with less noticeable results in the evening. The current results also indicate that trained subjects supplementing with caffeine in the evening did not benefit from caffeine. Because of the potential detrimental effects that evening caffeine consumption has on sleep, we recommend that athletes confirm that caffeine is effective on an individual basis before using in the evening. The research on external factors that may alter how an individual performs with caffeine supplementation is still sparse, and more information is needed before personalized prescription for optimal performance outcomes can be provided.

## Figures and Tables

**Table 1:** Descriptive Data for All Subjects and the Upper and Lower Cycle Training Tertiles.

	All Subjects (*n* = 20)	Trained (*n* = 7)	Untrained (*n* = 7)
Height (m)	1.75 ± 0.07	1.75 ± 0.07	1.76 ± 0.08
Body Mass (kg)	73.6 ± 10.9	70.2 ± 10.7	76.0 ± 10.6
Age (year)	22 [18–44]	22 [18–39]	21 [19–44]
V̇O_2peak_ (mL·kg^−1^·min^−1^)	57.2 ± 9.3	64.8 ± 7.9	49.2 ± 5.6
Caffeine Intake (mg/day)	32 [0–407]	100 [8–407]	2 [0–204]
Cycle Training (h/week)	4.0 [1.5–10.0]	8.0 [5.0–10.0]	2.3 [1.5–3.5]
Resistance Training (h/week)	1.0 [0–22.5]	1.5 [0–22.5]	3.5 [0–9]

Age, caffeine intake, cycle training, and resistance training are expressed as medians [range] because data did not display a normal distribution. All other variables are expressed as means ± SD. VO_2peak_ and cycling volume were higher in Trained vs. Untrained (*p* < 0.05).

**Table 2:** Peak Muscle Strength Data.

Velocity	30 Degrees/s		120 Degrees/s		240 Degrees/s	
Time	AM	PM	AM	PM	AM	PM
**PLA**	192.7 ± 39.1	190.7 ± 38.7	171.3 ± 31.7	171.7 ± 29.5	154.6 ± 28.6	157.9 ± 29.9
**CAF**	194.1 ± 47.5	202.3 ± 41.8	171.3 ± 33.0	174.7 ± 29.2	158.4 ± 33.6	160.0 ± 26.1
**PLA vs. CAF**	0.9 ± 4.4	5.2 ± 3.6	−0.3 ± 3.5	1.3 ± 3.1	2.0 ± 3.1	0.8 ± 3.6
	(−0.3 ± 4.3)	(5.94 ± 3.5)	(−0.1 ± 3.3)	(1.9 ± 2.9)	(2.0 ± 2.9)	(1.8 ± 3.6)
	12/85/3	72/28/0	4/91/6	10/90/1	18/81/0	9/89/2
	Likely Trivial	Possible; *p* = 0.07	Likely Trivial	Likely Trivial	Likely Trivial	Likely Trivial
**AM vs. PM**	−4.3 ± 5.5 (−6.19 ± 5.4)		−1.6 ± 4.6 (−2.0 ± 4.3)		1.3 ± 4.6 (0.2 ± 4.5)	
	1/46/53; Possible; *p* = 0.06		3/75/22; Likely Trivial		19/77/4; Likely Trivial	

Values for Placebo (PLA) and Caffeine (CAF) reported as Mean ± SD. AM, morning; PM, afternoon. Comparison values reported as adjusted (actual in parenthesis). Mean ± 90% CI for differences between change scores (i.e., AM vs. PM), % likelihoods of positive effect/trivial effect/negative effect and semantic inferences.
